# Correction: Water Spinach, *Ipomoea aquatica* (Convolvulaceae), Ameliorates Lead Toxicity by Inhibiting Oxidative Stress and Apoptosis

**DOI:** 10.1371/journal.pone.0143766

**Published:** 2015-11-24

**Authors:** The PLOS ONE Staff

## Notice of Republication

This article was republished on November 6, 2015, to correct the name of the aquatic plant in the title and the citation from “*Ipomoea aquatic*” to “*Ipomoea aquatica*.” The publisher apologizes for the error. Please download this article again to view the correct version. The originally published, uncorrected article and the republished, corrected articles are provided here for reference.

## Supporting Information

S1 File Originally published, uncorrected article. (PDF)

S2 File Republished, corrected article. (PDF)
