# Supplementary material for: Water Spinach, Ipomoea aquatica (Convolvulaceae), Ameliorates Lead Toxicity by Inhibiting Oxidative Stress and Apoptosis
Source: PLoS One. 2015 Oct 16;10(10):e0139831. doi: 10.1371/journal.pone.0139831 (PMC4608788; doi:10.1371/journal.pone.0139831)
Supplement: S1 Table — (DOCX) [file pone.0139831.s001.docx]

**S1 Table. Effect on antioxidant parameters in absence (Pb-acetate) and presence of AEIA (AEIA + Pb-acetate) in isolated mice hepatocytes.**

| **Parameters** | **Control** | **Pb-acetate**  **(6.8 µM)** | **AEIA (400 µg/ml) +**  **Pb-acetate (6.8 µM)** |
| --- | --- | --- | --- |
| **TBARS (µg/g of tissue** | 4.1 ± 0.2 | 6.9 ± 0.8^#^ | 5.2 ± 0.7^**^ |
| **Protein carbonyl (nmol/mg of protein)** | 45.9 ± 3.2 | 77.2 ± 4.3^#^ | 57.9 ± 3.9^*^ |
| **CAT (U/mg of protein)** | 244.2 ± 7.3 | 179.2 ± 6.2^#^ | 212.7 ± 7.8^*^ |
| **SOD (U/mg of protein)** | 84.5 ± 4.6 | 56.7 ± 3.5^#^ | 74.7 ± 4.7^*^ |
| **GST (µmol/min/mg protein)** | 68.4 ± 3.3 | 32.4 ± 2.1^#^ | 60.6 ± 2.3^**^ |
| **GPx (nmol/min/mg of protein)** | 75.4 ± 3.2 | 51.7 ± 3.5^#^ | 66.7 ± 3.4^*^ |
| **GR (nmol/min/mg of protein)** | 82.0 ± 3.1 | 54.7 ± 3.7^#^ | 67.7 ± 2.1^*^ |
| **GSH (nmol/mg of protein)** | 5.5 ± 0.4 | 3.3 ± 0.2^#^ | 4.6 ± 0.2^*^ |

Values are expressed as mean ± SE (n = 3). ^#^ Values differ significantly from normal control (p < 0.01). ^*^ Values differ significantly from Pb-acetate control (p < 0.05). ^**^Values differ significantly from Pb-acetate control (p < 0.01). CAT unit, ‘U’ is defined as µmoles of H_2_O_2_ consumed per minute. SOD unit, ‘U’ is defined as the µmoles inhibition of NBT reduction per minute.
