# Supplementary material for: Water Spinach, Ipomoea aquatica (Convolvulaceae), Ameliorates Lead Toxicity by Inhibiting Oxidative Stress and Apoptosis
Source: PLoS One. 2015 Oct 16;10(10):e0139831. doi: 10.1371/journal.pone.0139831 (PMC4608788; doi:10.1371/journal.pone.0139831)
Supplement: S2 Table — (DOCX) [file pone.0139831.s002.docx]

**S2 Table. Effect of AEIA (100 mg/kg, p.o.) on haematological parameters of experimental mice.**

| **Groups** | **Haematological and serum biochemical parameters** | **Values** |
| --- | --- | --- |
| Normal control | Total erythrocyte count (x10^6/^mm^3^) | 5.5 ± 0.4 |
| AEIA |  | 5.6 ± 0.3 |
| Normal control | Haemoglobin (g/dl) | 8.9 ± 0.7 |
| AEIA |  | 9.0 ± 0.5 |
| Normal control | Total leucocyte count (x10^3/^mm^3^) | 6.0 ± 0.6 |
| AEIA |  | 5.9 ± 0.3 |
| Normal control | Lactate dehydrogenase (U/l) | 29.2 ± 2.0 |
| AEIA |  | 30.3 ± 1.6 |
| Normal control | Creatinine kinase (IU/ mg protein) | 187.1 ± 11.5 |
| AEIA |  | 172.3 ± 18.5 |
| Normal control | Cholesterol (mg/dl) | 181.5 ± 12.0 |
| AEIA |  | 179.5 ± 11.0 |
| Normal control | Triglycerides (mg/dl) | 112.3 ± 4.2 |
| AEIA |  | 116.5 ± 8.1 |

Values are expressed as mean ± SE, for ten animals in each group. No significant difference was observed between two groups.
