# Supplementary material for: Water Spinach, Ipomoea aquatica (Convolvulaceae), Ameliorates Lead Toxicity by Inhibiting Oxidative Stress and Apoptosis
Source: PLoS One. 2015 Oct 16;10(10):e0139831. doi: 10.1371/journal.pone.0139831 (PMC4608788; doi:10.1371/journal.pone.0139831)
Supplement: S3 Table — (DOCX) [file pone.0139831.s003.docx]

**S3 Table. Effect of AEIA (100 mg/kg, p.o.) on ROS production, lipid peroxidation, protein carbonylation, antioxidant enzymes and GSH levels in liver, kidney, heart, brain and testes of experimental mice.**

| Parameters | Groups | Liver | Kidney | Heart | Brain | Testes |
| --- | --- | --- | --- | --- | --- | --- |
| ROS production  (nmol DCF/min/ mg of protein) | Normal | 20.3 ± 1.6 | 25.3 ± 1.2 | 20.7 ± 1.9 | 22.5 ± 2.1 | 21.3 ± 1.1 |
|  | AEIA | 21.5 ± 1.2 | 22.2 ± 1.3 | 20.4 ± 2.0 | 21.4 ± 1.7 | 20.9 ± 1.9 |
| Lipid peroxidation  (TBARS level in μg/g of tissue) | Normal | 5.1 ± 0.5 | 5.9 ± 0.3 | 5.7 ± 0.6 | 3.7 ± 0.2 | 4.1 ± 0.2 |
|  | AEIA | 5.3 ± 0.2 | 5.3 ± 0.4 | 5.7 ± 0.2 | 3.6 ± 0.5 | 4.3 ± 0.6 |
| Protein cabonylation  (nmol/mg of protein) | Normal | 32.3 ± 0.9 | 22.4 ± 1.0 | 12.7 ± 0.9 | 9.3 ± 0.7 | 9.0 ± 0.4 |
|  | AEIA | 34.1 ± 1.4 | 21.3 ± 1.4 | 11.2 ± 0.6 | 9.1 ± 0.4 | 9.6 ± 0.4 |
| CAT  (U/mg of protein) | Normal | 208.7 ± 19.7 | 219.0 ± 14.4 | 302.2 ± 20.3 | 104.1 ± 7.6 | 44.1 ± 3.3 |
|  | AEIA | 202.5 ± 12.9 | 225.2 ± 17.3 | 313.4 ± 15.0 | 103.4 ± 8.4 | 42.9 ± 2.1 |
| SOD  (U/mg of protein) | Normal | 117.3 ± 4.9 | 82.1 ± 4.2 | 104.1 ± 5.7 | 80.5 ± 3.3 | 89.7 ± 4.1 |
|  | AEIA | 116.5 ± 4.2 | 76.0 ± 3.7 | 110.0 ± 6.3 | 75.8 ± 3.9 | 89.1 ± 3.5 |
| GPx  (nmol/min/mg of protein) | Normal | 101.6 ± 5.8 | 65.4 ± 2.9 | 178.1 ± 16.0 | 145.1 ± 9.2 | 141.2 ± 7.2 |
|  | AEIA | 103.5 ± 6.1 | 64.2 ± 3.6 | 183.4 ± 14.7 | 147.5 ± 11.2 | 142.9 ± 9.6 |
| GR  (nmol/min/mg of protein) | Normal | 75.1 ± 4.1 | 59.4 ± 3.1 | 72.1 ± 3.9 | 49.2 ± 2.9 | 77.0 ± 3.8 |
|  | AEIA | 71.9 ± 3.2 | 60.1 ± 2.3 | 73.9 ± 3.7 | 50.4 ± 2.5 | 76.9 ± 4.1 |
| GST  (µmol/min/mg of protein) | Normal | 1.1 ± 0.2 | 1.2 ± 0.1 | 0.9 ± 0.05 | 1.2 ± 0.1 | 1.7 ± 0.2 |
|  | AEIA | 1.3 ± 0.08 | 1.1 ± 0.09 | 1.0 ± 0.04 | 1.2 ± 0.04 | 1.8 ± 0.3 |
| GSH  (nmol/mg protein) | Normal  AEIA | 26.9 ± 1.4  27.8 ± 0.9 | 24.0 ± 0.8  22.8 ± 1.1 | 24.8 ± 1.1  26.1 ± 0.8 | 20.1 ± 1.0  21.5 ± 1.4 | 20.6 ± 1.5  19.9 ± 0.9 |

Values are expressed as mean ± SE, for ten animals in each group. No significant difference was observed between two groups.
