# Supplementary material for: Water Spinach, Ipomoea aquatica (Convolvulaceae), Ameliorates Lead Toxicity by Inhibiting Oxidative Stress and Apoptosis
Source: PLoS One. 2015 Oct 16;10(10):e0139831. doi: 10.1371/journal.pone.0139831 (PMC4608788; doi:10.1371/journal.pone.0139831)
Supplement: S4 Table — (DOCX) [file pone.0139831.s004.docx]

**S4 Table. Effect on antioxidant enzymes and GSH levels in liver, kidney, heart, brain and testes in absence (Pb-acetate) and presence of AEIA (AEIA + Pb-acetate) in mice.**

| **Parameters** | **Group** | **Liver** | **Kidney** | **Heart** | **Brain** | **Testes** |
| --- | --- | --- | --- | --- | --- | --- |
| **Pb concentration**  **(µg/g of wet tissue)** | I | 0.1 ± 0.003 | 0.2 ± 0.003 | 0.1 ± 0.001 | 0.08 ± 0.001 | 0.1 ± 0.002 |
|  | II | 3.1 ± 0.09^#^ | 3.6 ± 0.07^#^ | 2.1 ± 0.04^#^ | 2.2 ± 0.09^#^ | 3.0 ± 0.04^#^ |
|  | III | 1.7 ± 0.04^**^ | 1.9 ± 0.03^**^ | 1.3 ± 0.03^**^ | 1.8 ± 0.03^**^ | 2.1 ± 0.02^**^ |
| **DNA fragmentation**  **(% over control)** | I | 100.0 ± 1.0 | 100.0 ± 1.0 | 100.0 ± 1.9 | 100.0 ± 1.6 | 100.0 ± 1.4 |
|  | II | 145.7 ± 3.1^#^ | 148.3 ± 3.7^#^ | 148.3 ± 3.2^#^ | 139.8 ± 3.8^#^ | 142.3 ± 3.0^#^ |
|  | III | 119.3 ± 2.7^**^ | 114.3 ± 3.2^**^ | 116.1 ± 3.1^**^ | 126.5 ± 3.2^*^ | 119.2 ± 2.7^**^ |
| **ATP**  **(nmol/mg of protein)** | I | 14.8 ± 1.1 | 22.4 ± 1.0 | 17.6 ± 0.6 | 11.1 ± 1.1 | 17.3 ± 1.1 |
|  | II | 27.7 ± 2.1^#^ | 35.3 ± 1.7^#^ | 29.3 ± 1.2^#^ | 19.3 ± 1.3^#^ | 26.2 ± 2.0^#^ |
|  | III | 20.3 ± 1.8^*^ | 24.3 ± 1.2^**^ | 19.1 ± 1.1^**^ | 14.1 ± 1.1^*^ | 18.2 ± 0.8^**^ |

Values are expressed as mean ± SE, for six animals in each group. ^#^ Values differ significantly from normal control (p < 0.01). ^*^ Values differ significantly from Pb-acetate control (p < 0.05). ^**^Values differ significantly from Pb-acetate control (p < 0.01).
