# Supplementary material for: Water Spinach, Ipomoea aquatica (Convolvulaceae), Ameliorates Lead Toxicity by Inhibiting Oxidative Stress and Apoptosis
Source: PLoS One. 2015 Oct 16;10(10):e0139831. doi: 10.1371/journal.pone.0139831 (PMC4608788; doi:10.1371/journal.pone.0139831)
Supplement: S5 Table — (DOCX) [file pone.0139831.s005.docx]

**S5 Table. Effect on ROS production, lipid peroxidation, protein carbonylation and Co-enzymes Qs levels in liver, kidney, heart and brain in absence (Pb-acetate) and presence of AEIA (AEIA + (Pb-acetate) in mice.**

| **Parameters** | **Gr** | **Liver** | **Kidney** | **Heart** | **Brain** | **Testes** |
| --- | --- | --- | --- | --- | --- | --- |
| **ROS production**  **(nmol DCF/min/ mg of protein)** | I | 22.6 ± 1.7 | 27.1 ± 2.2 | 21.2 ± 1.2 | 23.5 ± 1.3 | 20.5 ± 1.7 |
|  | II | 58.6 ± 3.9^#^ | 65.3 ± 3.1^#^ | 51.1 ± 2.3^#^ | 47.1 ± 2.8^#^ | 54.3 ± 2.4^#^ |
|  | III | 28.5 ± 2.7^**^ | 35.4 ± 2.0^**^ | 31.2 ± 3.0^**^ | 29.3 ± 2.0^**^ | 32.2 ± 2.0^**^ |
| **Lipid peroxidation**  **(TBARS level in μg/g of tissue)** | I | 5.2 ± 0.7 | 6.5 ± 0.7 | 5.1 ± 0.3 | 3.7 ± 0.3 | 4.1 ± 0.5 |
|  | II | 8.6 ± 0.9^#^ | 11.4 ± 1.1^#^ | 8.0 ± 0.7^#^ | 5.6 ± 0.5^#^ | 6.9 ± 0.7^#^ |
|  | III | 6.0 ± 0.5^*^ | 8.3 ± 0.8^*^ | 5.6 ± 0.3^**^ | 4.0 ± 0.4^*^ | 4.7 ± 0.6^*^ |
| **Protein cabonylation**  **(nmol/mg of protein)** | I | 32.8 ± 1.9 | 20.4 ± 0.5 | 11.7 ± 0.7 | 9.1 ± 0.6 | 9.3 ± 0.5 |
|  | II | 52.7 ± 3.1^#^ | 44.3 ± 1.7^#^ | 23.3 ± 1.2^#^ | 17.8 ± 0.8^#^ | 19.2 ± 1.0^#^ |
|  | III | 34.3 ± 2.8^**^ | 28.3 ± 1.2^**^ | 14.1 ± 1.1^**^ | 13.5 ± 0.4^**^ | 11.2 ± 0.8^**^ |
| **Total coenzyme Q9**  **(nmol/g of wet tissue)** | I | 132.3 ± 7.7 | 139.3 ± 7.0 | 65.4 ± 4.2 | 30.2 ± 2.2 | 61.2 ± 4.0 |
|  | II | 99.3 ± 6.3^#^ | 101.9 ± 5.5^#^ | 42.2 ± 3.4^#^ | 22.7 ± 1.2^$^ | 42.5 ± 3.4^#^ |
|  | III | 125.7 ± 6.7^*^ | 126.4 ± 5.9^*^ | 58.1 ± 3.9^*^ | 28.7 ± 1.5^*^ | 56.0 ± 3.7^*^ |
| **Total coenzyme Q10**  **(nmol/g of wet tissue)** | I | 27.1 ± 1.9 | 24.5 ± 1.3 | 31.8 ± 2.0 | 12.2 ± 1.0 | 17.0 ± 1.0 |
|  | II | 14.5 ± 1.1^#^ | 15.3 ± 1.2^#^ | 17.3 ± 1.3^#^ | 8.0 ± 0.5^#^ | 11.5 ± 0.8^#^ |
|  | III | 24.7 ± 1.9^**^ | 22.3 ± 1.3^**^ | 27.6 ± 1.3^*^ | 10.5 ± 0.5^*^ | 15.5 ± 1.1^*^ |

Values are expressed as mean ± SE, for six animals in each group. ^#^ Values differ significantly from normal control (p < 0.01). ^$^ Values differ significantly from normal control (p < 0.01). ^*^ Values differ significantly from Pb-acetate control (p < 0.05). ^**^Values differ significantly from Pb-acetate control (p < 0.01).
