# Supplementary material for: Water Spinach, Ipomoea aquatica (Convolvulaceae), Ameliorates Lead Toxicity by Inhibiting Oxidative Stress and Apoptosis
Source: PLoS One. 2015 Oct 16;10(10):e0139831. doi: 10.1371/journal.pone.0139831 (PMC4608788; doi:10.1371/journal.pone.0139831)
Supplement: S6 Table — (DOCX) [file pone.0139831.s006.docx]

**S6 Table. Effect on antioxidant enzymes and GSH levels in liver, kidney, heart, brain and testes in absence (Pb-acetate) and presence of AEIA (AEIA + Pb-acetate) in mice.**

| **Parameters** | **Group** | **Liver** | **Kidney** | **Heart** | **Brain** | **Testes** |
| --- | --- | --- | --- | --- | --- | --- |
| **CAT**  **(U/mg of protein)** | I | 212.9 ± 15.3 | 216.4 ± 21.4 | 313.2 ± 21.3 | 94.1 ± 6.7 | 41.25 ± 3.9 |
|  | II | 132.3 ± 12.7^#^ | 122.3 ± 14.2^#^ | 192.6 ± 17.0^#^ | 46.5 ± 3.9^#^ | 25.54 ± 3.0^#^ |
|  | III | 203.1 ± 17.7^**^ | 194.3 ± 19.8^*^ | 267.5 ± 16.9^*^ | 69.8 ± 6.8^*^ | 38.76 ± 3.0^*^ |
| **SOD**  **(U/mg of protein)** | I | 112.3 ± 7.7 | 87.5 ± 6.0 | 101.3 ± 9.1 | 87.9 ± 5.7 | 94.3 ± 5.9 |
|  | II | 76.5 ± 5.4^#^ | 50.1 ± 4.9^#^ | 60.5 ± 5.4^#^ | 56.2 ± 4.0^#^ | 58.6 ± 4.7^#^ |
|  | III | 99.0 ± 6.1^*^ | 80.0 ± 6.5^**^ | 92.7 ± 8.3^**^ | 79.7 ± 6.0^*^ | 86.7 ± 4.9^**^ |
| **GPx**  **(nmol/min/mg of protein)** | I | 112.6 ± 6.2 | 60.2 ± 4.0 | 198.4 ± 15.1 | 142.3 ± 6.2 | 153.3 ± 8.8 |
|  | II | 64.1 ± 4.4^#^ | 44.1 ± 3.3^$^ | 124.2 ± 12.3^#^ | 110.1 ± 4.3^#^ | 101.1 ± 5.3^#^ |
|  | III | 101.6 ± 6.1^**^ | 59.9 ± 4.1^*^ | 172.5 ± 13.0^*^ | 131.0 ± 5.9^*^ | 145.8 ± 9.1^**^ |
| **GR**  **(nmol/min/mg of protein)** | I | 78.2 ± 6.1 | 58.1 ± 2.1 | 72.2 ± 4.5 | 45.2 ± 3.2 | 77.4 ± 4.0 |
|  | II | 49.2 ± 4.1^#^ | 27.9 ± 1.4^#^ | 34.3 ± 1.9^#^ | 26.7 ± 1.3^#^ | 42.2 ± 3.5^#^ |
|  | III | 71.5 ± 6.8^*^ | 51.3 ± 2.8^**^ | 66.6 ± 3.0^**^ | 36.0 ± 2.3^*^ | 75.3 ± 3.9^**^ |
| **GST**  **(µmol/h/mg of protein)** | I | 1.2 ± 0.1 | 1.1 ± 0.1 | 0.9 ± 0.02 | 1.6 ± 0.1 | 2.9 ± 0.3 |
|  | II | 0.6 ± 0.07^#^ | 0.5 ± 0.09^#^ | 0.6 ± 0.01^#^ | 1.1 ± 0.1^#^ | 1.2 ± 0.1^#^ |
|  | III | 1.1 ± 0.07^**^ | 0.9 ± 0.08^*^ | 0.8 ± 0.02^**^ | 1.5 ± 0.09^*^ | 2.5 ± 0.3^**^ |
| **GSH**  **(nmol/mg protein)** | I | 27.4 ± 2.1 | 23.2 ± 1.5 | 24.2 ± 1.8 | 21.3 ± 1.3 | 19.5 ± 1.0 |
|  | II | 15.5 ± 1.3^#^ | 14.2 ± 1.2^#^ | 14.1 ± 1.2^#^ | 13.7 ± 1.4^#^ | 11.8 ± 1.0^#^ |
|  | III | 23.3 ± 1.8^**^ | 19.3 ± 1.4^*^ | 21.1 ± 1.9^*^ | 18.7 ± 1.1^*^ | 16.1 ± 1.2^*^ |

Values are expressed as mean ± SE, for six animals in each group. ^#^ Values differ significantly from normal control (p < 0.01). ^*^ Values differ significantly from Pb-acetate control (p < 0.05). ^**^Values differ significantly from Pb-acetate control (p < 0.01).
