# Supplementary material for: Correction: Water Spinach, Ipomoea aquatica (Convolvulaceae), Ameliorates Lead Toxicity by Inhibiting Oxidative Stress and Apoptosis
Source: PLoS One. 2015 Nov 24;10(11):e0143766. doi: 10.1371/journal.pone.0143766 (PMC4658196; doi:10.1371/journal.pone.0143766)
Supplement: S2 File — (PDF) [file pone.0143766.s002.pdf]

PLOS | ONE

RESEARCH ARTICLE

Water Spinach, *Ipomoea aquatica* (Convolvulaceae), Ameliorates Lead Toxicity by Inhibiting Oxidative Stress and Apoptosis

Saikat Dewanjee[1]*, Tarun K. Dua[1], Ritu Khanra[1], Shilpa Das[1], Sujata Barma[1], Swarnalata Joardar[1], Niloy Bhattacharjee[1], M. Zia-Ul-Haq[2], Hawa Z. E. Jaafar[3]*

1 Advanced Pharmacognosy Research Laboratory, Department of Pharmaceutical Technology, Jadavpur University, Kolkata, 700032, India, **2** Department of Pharmacognosy, University of Karachi, Karachi, 75270, Pakistan, **3** Department of Crop Science, Faculty of Agriculture, Universiti Putra, Selangor, 43400, Malaysia

* s.dewanjee@yahoo.com (SD); hawazej@gmail.com (HZEJ)

CrossMark
click for updates

OPEN ACCESS

Citation: Dewanjee S, Dua TK, Khanra R, Das S, Barma S, Joardar S, et al. (2015) Water Spinach, *Ipomoea aquatica* (Convolvulaceae), Ameliorates Lead Toxicity by Inhibiting Oxidative Stress and Apoptosis. PLoS ONE 10(10): e0139831. doi:10.1371/journal.pone.0139831

Editor: Hemachandra Reddy, Texas Tech University Health Science Centers, UNITED STATES

Received: July 13, 2015

Accepted: September 17, 2015

Published: October 16, 2015

Copyright: © 2015 Dewanjee et al. This is an open access article distributed under the terms of the Creative Commons Attribution License, which permits unrestricted use, distribution, and reproduction in any medium, provided the original author and source are credited.

Data Availability Statement: All relevant data are within the paper and its Supporting Information files.

Funding: This study was supported by the Council for Scientific and Industrial Research-senior research fellowship Ref. No.: 09/096(773)/2013-EMR-I dated 15th March, 2013.

Competing Interests: The authors have declared that no competing interests exist.

Abstract

Background

Ipomoea aquatica (Convolvulaceae), an aquatic edible plant, is traditionally used against heavy metal toxicity in India. The current study intended to explore the protective role of edible (aqueous) extract of *I. aquatica* (AEIA) against experimentally induced Pb-intoxication.

Methods

The cytoprotective role of AEIA was measured on mouse hepatocytes by cell viability assay followed by Hoechst staining and flow cytometric assay. The effect on ROS production, lipid peroxidation, protein carbonylation, intracellular redox status were measured after incubating the hepatocytes with Pb-acetate (6.8 μM) along with AEIA (400 μg/ml). The effects on the expressions of apoptotic signal proteins were estimated by western blotting. The protective role of AEIA was measured by *in vivo* assay in mice. Haematological, serum biochemical, tissue redox status, Pb bioaccumulation and histological parameters were evaluated to estimate the protective role of AEIA (100 mg/kg) against Pb-acetate (5 mg/kg) intoxication.

Results

Pb-acetate treated hepatocytes showed a gradual reduction of cell viability dose-dependently with an IC_{50} value of 6.8 μM. Pb-acetate treated hepatocytes exhibited significantly enhanced levels ($p < 0.01$) of ROS production, lipid peroxidation, protein carbonylation with concomitant depletion ($p < 0.01$) of antioxidant enzymes and GSH. However, AEIA treatment could significantly restore the aforementioned parameters in murine hepatocytes near to normalcy. Besides, AEIA significantly reversed ($p < 0.05$–0.01) the alterations of transcription levels of apoptotic proteins viz. Bcl 2, Bad, Cyt C, Apaf-1, cleaved caspases [caspase 3, caspase 8 and caspase 9], Fas and Bid. In *in vivo* bioassay, Pb-acetate treatment caused significantly high intracellular Pb burden and oxidative pressure in the kidney, liver, heart, brain and testes in mice. In addition, the haematological and serum biochemical

factors were changed significantly in Pb-acetate-treated animals. AEIA treatment restored significantly the evaluated-parameters to the near-normal position.

Conclusion

The extract may offer the protective effect via counteracting with Pb mediated oxidative stress and/or promoting the elimination of Pb by chelating. The presence of substantial quantities of flavonoids, phenolics and saponins would be responsible for the overall protective effect.

Introduction

Lead (Pb), a ubiquitous industrial and environmental pollutant, play a major role in pathogenesis of mammalian cells by unsettling the critical pro-oxidant to antioxidant ratio. The amount of Pb utilized in the present era is much higher than total amount consumed in earlier centuries [1]. Pb is known to induce a broad spectrum pathogenesis in including haemopoietic system [2], livers [3], kidneys [4], brains [5], hearts [6] and reproductive systems [7]. After absorption, Pb enters into the blood circulation and accumulates in erythrocytes. Then it is distributed in many organs, preferentially to livers and kidneys. After accumulation within the organs, Pb causes reactive oxygen species (ROS) production and binds with functional -SH groups. Excessive production of ROS can damage oxidatively the cellular macromolecules and induce apoptotic events. On the other hand, binding of Pb with functional -SH groups of enzymes subsequently renders the enzymes nonfunctional and depresses their activities. Due to strong links between oxidative stress and exposure to Pb, attention is being paid to utilize the shielding effects of natural antioxidants against chemically induced Pb toxicity [8].

Water spinach, *Ipomoea aquatica* Forssk. (Convolvulaceae), is an aquatic or semi-aquatic edible herb [9]. *I. aquatica* is used traditionally against various disorders like diabetes, liver malfunction, constipation and in the treatment of arsenic and heavy metal poisoning [9,10]. Literature reviews revealed the occurrence of significant amounts of phenolic compounds, flavonoids, saponins, β-carotene and ascorbic acid in *I. aquatica* [9]. The current study has been designed to determine the role of aqueous extract of *I. aquatica* against experimentally induced Pb toxicity. The effect of Pb alone and protective effect of *I. aquatica* extract were measured employing suitable *in vitro* (on mouse hepatocytes) and *in vivo* (on experimental mice) models. Significant attempts were made to elucidate the mechanism of actions against Pb-intoxication and to establish the correlation between observed effects with the phytochemicals present within the test material.

Materials and Methods

Chemicals

The chemicals utilized during study: ammonium sulphate, 2,4-dinitro-phenyl-hydrazine, 1-chloro-2,4-dinitrobenzene, ethylenediaminetetraacetic acid, 5,5-di-thio-bi(2-nitrobenzoic acid), N-ethylmaleimide, nitro blue tetrazolium, reduced nicotinamide adenine dinucleotide, potassium dihydrogen phosphate, phenazinemethosulphate, sodium pyrophosphate, reduced glutathione, sodium azide, thiobarbituric acid, 5-thio-2-nitrobenzoic acid and trichloro acetic acid were procured from Sisco Research Laboratory, India. Bovine serum albumin, Bradford reagent and Pb-acetate were purchased from Sigma-Aldrich, St. Louis, USA. All primary

antibodies (produced in rabbit) viz. anti-Bcl-2 (dilution 1:1000), anti-Bad (dilution 1:3000), anti-Cyt C (dilution 1:1000), anti-Apaf-1 (dilution 1:1000), anti-caspase 3 (dilution 1:1000), anti-caspase 8 (dilution 1:1000), anti-caspase 9 (dilution 1:1000), anti-Fas (dilution 1:2000), anti-Bid (dilution 1:1000) and anti-actin (dilution 1:3000) for immunoblotting were purchased from Sigma-Aldrich Chemical Company St. Louis, USA. Appropriate HRP conjugated secondary antibody (dilution 1:3000) produced in goat was also purchased from Sigma-Aldrich Chemical Company St. Louis, USA. Methanol, formic acid, acetic acid and acetonitrile were obtained from Merck, India.

Preparation of extract

The aqueous extract (AIEA) was prepared by macerating the dried and powdered arial part of *I. aquatica* with distilled water containing 1% of chloroform for 48 h at 30 ± 5°C with continuous stirring [9]. The resultant extract was filtered and lyophilized to yield the powdered crude extracts AEIA (~11.7%, w/w). To prepare the samples, lypholized powder was dissolved in distilled water containing 1% tween 80 prior to *in vivo* experiment. For *in vitro* assays, the extract was solubilized in DMSO and diluted with autoclaved distilled water into desired concentrations (resultant $\leq$ 0.4% DMSO in contact to cells).

Phytochemical analysis

Phytochemical analysis revealed substantial quantities of flavonoids (~37.9 mg/g^{DW}), phenolics (~22.7 mg/g^{DW}), saponins (~51.2 mg/g^{DW}), carbohydrates (~132.7 mg/g^{DW}) and ascorbic acid (~3.1 mg/g^{DW}) [9]. RP-HPLC analysis revealed presence of myricetin, quercetin and apigenin in AEIA [9].

Animals

Healthy Swiss albino mice (♂, 25 ± 5 g) used in this study were maintained under standard lab conditions. The principles of laboratory animal care (Public Health Service, 1986) were followed throughout the experiment [11]. The animal ethics committee (AEC) (Ref. no. 0367/01/C/cpcsea), Jadavpur University approved the experiments on animals (Ref no. AEC/PHARM/1501/02/2015 dated 18.03.2015) for this study. The instructions given by Govt. of India and university grants commission (UGC), India for the experiments with laboratory animals were followed throughout the experiment.

In vitro bio-assays

Hepatocyte isolation and culture. The mice were subjected to CO_2 euthanasia and sacrificed by cervical dislocation. The livers were excised and rinsed with PBS (pH 7.4). Hepatocytes were isolated from mouse liver by the collagenase perfusion method mentioned by Sarkar et al [12] with little modifications. Hepatocytes were filtered from a syringe (wide-bore), and centrifuged for 5 min (500 rpm). The resulting pellet was suspended again in DMEM (FBS 10%) and reared at 5% CO_2 and 37°C to achieve monolayer in the culture flask. The hepatocytes were passaged at least twice before *in vitro* assays.

Determination of cytotoxic effect of Pb-acetate. Concentration dependent cytotoxic effect of Pb-acetate was determined by cell viability assay. Briefly, different groups of hepatocyte cultures (~2×10^6 cells/culture) were incubated with Pb-acetate (0.25–10000 μM) at 37°C and 5% CO_2 tension for 2 h. MTT assay was used to count the sustainable cells percentage [13].

Assessment of cytoprotective role of AEIA. A cell viability assay has been performed to measure cytoprotective role of AEIA against Pb-acetate induced cytotoxicity. Briefly, different sets of hepatocytes (~2×10^6 cells/set) were incubated at 37°C with Pb-acetate (6.8 μM, IC_{50}) and Pb-acetate (6.8 μM) along with extracts (50, 100, 200 and 400 μg/ml) for different times up to 4 h. MTT assay was used to count the sustainable cells percentage [13]. Two sets with and without Pb-acetate was kept as toxic and normal control groups, respectively.

Hoechst staining. Hoechst staining was performed to detect the nucleus within viable cells under fluorescence microscope [14]. Briefly, tissue culture plates were utilized to obtain 2000 cells per well in 384 well plate and incubated at 37°C and 5% CO_2. 24 h after seeding, the cells were incubated with Pb-acetate (6.8 μM) + AEIA (400 μg/ml). 2 h later, the para-formal-dehyde (4%) was used to fix the cells for 25 minutes. The fixed-cells were incubated with Hoechst 33258 (5 μg/ml in PBS) for 15 min. Fluorescent nuclei were scored as an index of cell viability. Two sets with and without Pb-acetate were kept as toxic and normal control groups, respectively.

Flow Cytometric Analysis (FCA). To assess the nature of death, FCA was performed in the current study. Briefly, hepatocytes were incubated with Pb-acetate (6.8 μM) + AEIA (400 μg/ml) for 2 h at 37°C and 5% CO_2. Two sets with and without Pb-acetate were kept as toxic and normal control, respectively. The hepatocytes were treated with FITC-labelled annexin V and PI (propidium iodide) at 37°C for half an hour. After washing of extra Annexin V and PI; cells were fixed and analysed by flow cytometry by FACS-calibur (Laser light 488 nm argon; band pass filter of 515 nm and 623 nm for FITC fluorescence and PI fluorescence respectively) by using Cell-Quest software. A scatter plot of both fluorescences was prepared.

Assays of antioxidant markers. Various groups of hepatocytes, each comprising suspension (1 ml; ~ 2×10^6 cells/ml) were used in these experiments. The protective role of the AEIA against Pb-intoxication was analyzed by incubating hepatocytes with Pb-acetate (6.8 μM) + AEIA (400 μg/ml) at 37°C for 2 hrs [13]. Two sets with and without Pb-acetate were kept as toxic and normal control groups, respectively. Generation of intra-cellular ROS was quantified by the modified method described by Kim and co-workers [15] of original method developed by LeBel and Bondy [16] with little modifications made by Kim et al [16] and DCF-formation was measured by using a fluorescence spectrometer at the excitation and emission wavelengths of 488 and 510 nm, respectively. The magnitude of lipid peroxidation was measured as TBARS (thiobarbituric acid reactive substances) by the method of Ohkawa and co-workers [17] while Uchida and Stadtman protocol was followed to measure protein carbonylation [18]. The levels of antioxidant enzymes like CAT (catalase), SOD (superoxide dismutase), GR (glutathione reductase), GPx (glutathione peroxidase) and GST (glutathione-s-transferase) were evaluated by method of Ghosh et al [19]. GSH (reduced glutathione) level were measured by the method of Hissin and Hilf [20].

Immunoblotting of signaling proteins. The isolated proteins (20 μg) from different tested hepatocytes were exposed to SDS-PAGE (10% gel) and by standard dry transfer method, were transferred to a membrane (nitro-cellulose) [21]. Membranes were blocked (4°C; 1 h) in buffer (blocking) and were washed with TBST. Then primary anti-bodies were incubated with respective membranes overnight at 4°C. The TBST was used to rinse membranes again and incubated with suitable HRP-conjugated secondary anti-body at room temperature for 1 hr. The blots were generated by the HRP substrate system.

In vivo bioassay

Experimental design. After 14 days of adaptation to the lab conditions, the animals (n = 18) were divided into three groups (n = 6/per group) as:

Group I: normal control, received distilled (double) H_2O during whole experiments;

Group II: toxic control, received Pb-acetate aqueous solution, @ 5 mg/Kg for 40 days [22];

Group III: AEIA-treated mice @ 100 mg/Kg once daily for 30days from 11th day after beginning of Pb-acetate treatment prior to Pb-acetate administration @ 5 mg/kg [22].

On day 41, the mice were subjected to CO_2 euthanasia and sacrificed by cervical dislocation. Blood samples were collected from retro-orbital venous complex before sacrificing the animals. The organs were removed and rinsed with phosphate buffer saline (pH 7.4). The organs were homogenized in 0.1 M Tris-HCl-0.001 M EDTA buffer (pH 7.4) and centrifuged (12,000 g) at 4°C for half an hour. The supernatant obtained was collected and used for assessment of biochemical parameters.

Haematological parameters. The blood corpuscle counts and haemoglobin content were determined by haemocytometer and haemoglobinometer, respectively. Serum biochemical parameters viz. lactate dehydrogenase (LDH), creatinine kinase (CK), cholesterol and triglycerides levels, were evaluated by kits manufactured by Span Diagnostic Limited, India.

Assessment of antioxidant markers related to organ dysfunction. Distribution of Pb in tissues was measured by atomic absorption (flame) spectroscopy. The intercellular ROS, DNA fragmentation, TBARS level, antioxidant enzymes, and non-enzymatic antioxidant were assayed following standard assay protocols mentioned earlier. Co-enzymes Q (Q_9 and Q_{10}) were isolated and estimated according to the method of Zhang and co-workers [23]. Intracellular ATP concentration was assessed by commercial kits (Abcam, Cambridge, USA) following manufacturer protocol.

Histopathological studies. The organs of experimental and normal mice were fixed in formalin (10%) and were preceded for sectioning (paraffin). Sections (5μm) were stained by eosin and hematoxylin to report the histology [24]. Histo-quantification was performed using NIH IMAGE (Image-J, 1.37v) software by a person who was blinded to the experiment. Sixty random sections/group were investigated to quantify the histological parameters [25–31].

Statistical analysis

Data were analyzed by one-way ANOVA and reported as mean ± SE followed by Dunnett's t-test. The significance was marked only when $p < 0.05$.

Results and Discussion

Effect of AEIA against Pb-acetate-intoxication *in vitro*

Effect on cell viability *in vitro*. Cell viability indicates the degree of cytotoxity caused by any toxicant. Fig 1A depicted the cytotoxic effect of Pb-acetate on murine hepatocytes. Incubation of hepatocytes with Pb-acetate for 2 h exhibited a concentration dependent cytotoxic effect. The IC_{50} value was found to be ~ 6.8 μM. Based on observed IC_{50} value, Pb-acetate (6.8 μM) was used in the subsequent *in vitro* assays. The incubation of hepatocytes with AEIA (50–600 μg/ml) could significantly counteract with Pb-acetate induced cytotoxicity (Fig 1B). The extract could improve cell viability in a concentration dependent manner between 50–400 μg/ml, however, the cytoprotective effect was reduced beyond 400 μg/ml as observed at the dose of 600 μg/ml of AEIA. The reduction of cytoprotective effect beyond the dose of 400 μg/ml may be due to the prooxidative effects of AEIA at the concentration higher then optimal concentration require to exert physiological benefit. 'Double-edged sword' effect of exogenous antioxidants has been demonstrated in the literature [32]. Based on the observed effect AEIA (400 μg/ml) has been chosen for subsequent *in vitro* assays.

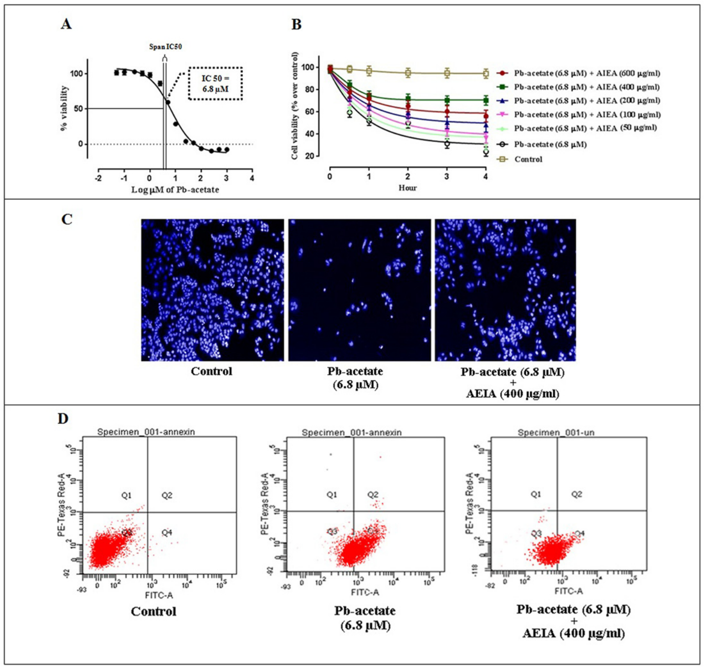

Fig 1. Cell viability studies in the absence (Pb-acetate) and existence of AEIA (Pb-acetate + AEIA) *in vitro*. Panel A. Effect of Pb-acetate at different concentrations in cell viability in mouse hepatocytes. Panel B. Concentration and time-dependent effect on cell viability studies in the absence (Pb-acetate) and existence of AEIA in isolated murine hepatocytes. Values are denoted as mean ± SE (n = 3). Panel C. Hoechst staining of murine hepatocytes in the absence (Pb-acetate) and existence of AEIA (Pb-acetate + AEIA). Panel D. Percentage distribution of apoptotic and necrotic cells in the absence (Pb-acetate) and existence of AEIA (Pb-acetate + AEIA) by FCA.

doi:10.1371/journal.pone.0139831.g001

To observe the cytotoxic effect of Pb-acetate and protective effect of AEIA, hepatocytes were subjected to Hoechst staining following visualization under fluorescence microscope (Fig 1C). Pb-acetate treated hepatocytes exhibited significantly less number of visible nuclei, however, the visible nuclei exhibited specific pattern of morphological changes. However, AEIA treatment could reinstate the viability of cells in image assay apparent from nuclear count.

To investigate the nature of cell death, hepatocytes were assessed by FCA. Flow cytometric data (Fig 1D) exhibited that Pb-treated hepatocytes showed very low PI staining (~ 0.2%) and very high annexin V-FITC binding (~ 55.5%) indicating majority of apoptotic cells. In AEIA (400 μg/ml)-treated hepatocytes, apoptotic cell figure was low significantly (~ 19.2%) indicating a possible anti-apoptotic role of AEIA against Pb-induced toxicity. While, control group showed very little apoptotic (~ 0.08%) and necrotic (~ 0.02%) cells as compared with viable cells (~ 99.1%).

In the earlier report, we have reported that the AEIA (400 μg/ml) alone does not possess any significant alteration of cell viability as compared with untreated hepatocytes [33].

Effect on ROS-generation, protein-carbonylation, lipid-peroxidation and redox status. Fig 2 represented the effect of AEIA against Pb-acetate induced alteration of ROS production, peroxidation of lipids, carbonylation of proteins and intracellular redox status in murine hepatocytes. The enhance production of intracellular ROS is a marker of augmented oxidative stress (Fig 2A). Generation of ROS (intracellular) was measured by microscopy (fluorescence) using fluorescent dye DCF. In this study, Pb-acetate exposure significantly increased intracellular ROS production in murine hepatocytes. The AEIA treatment could significantly

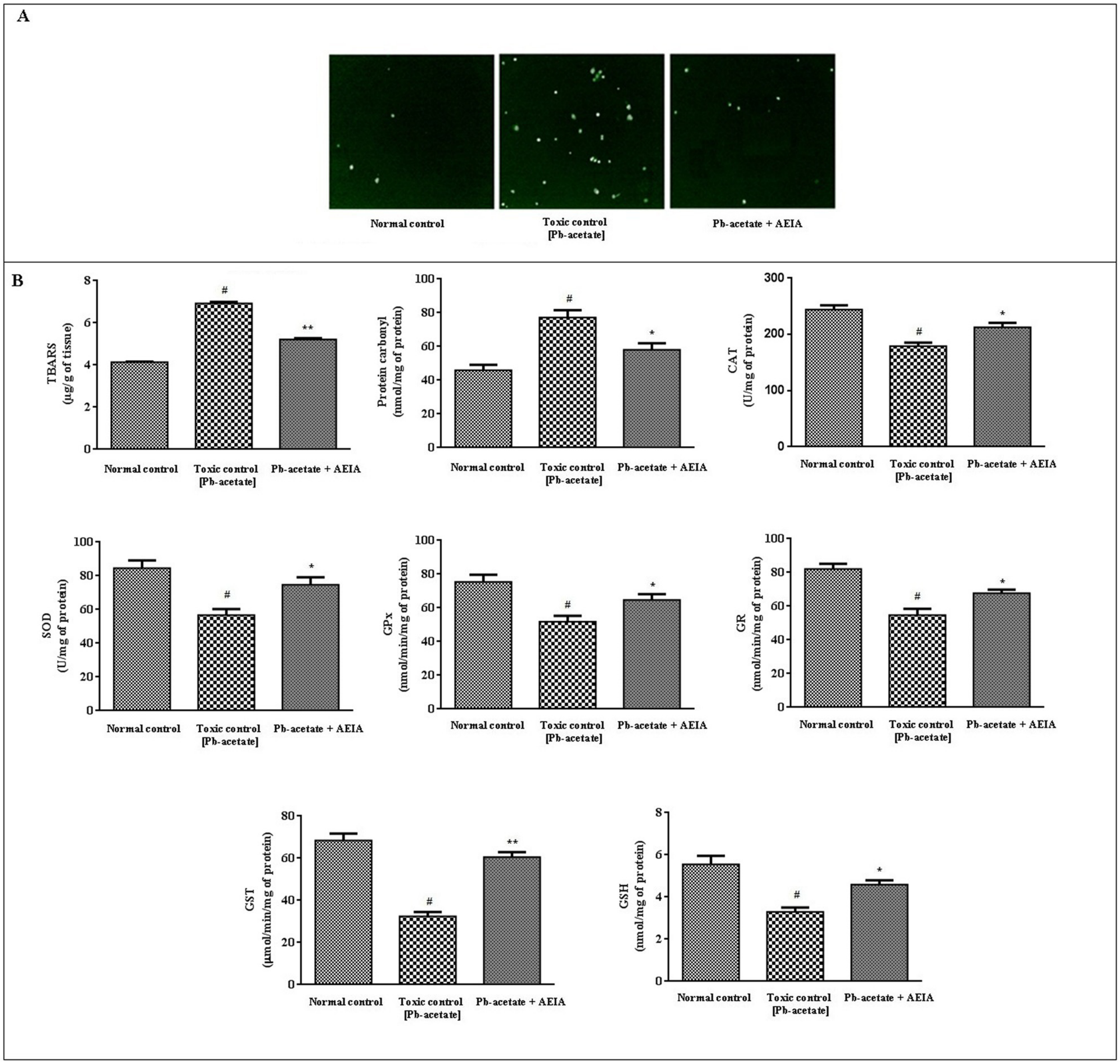

Fig 2. The effect on cellular redox markers in the absence (Pb-acetate) and existence of AEIA (Pb-acetate + AEIA) in murine hepatocytes. Panel A. Effect on ROS-generation (intrecellular) was measured by fluorescence microscopy (DCF-DA) in Pb-exposed hepatocytes in the absence (Pb-acetate) and existence of AEIA (Pb-acetate + AEIA). Panel B. Effect on antioxidant parameters viz. lipid peroxidation, protein carbonylation, SOD, CAT, GST, GPx, GSH and GR in the absence (Pb-acetate) and existence of AEIA (Pb-acetate + AEIA). Values are expressed as mean ± SE (n = 3). #Values differed significantly from normal control ($p < 0.01$). *Values differed significantly from pb-acetate control ($p < 0.05$). **Values differed significantly from pb-acetate control ($p < 0.01$). SOD unit, "U" is defined as µ-moles inhibition of NBT-reduction/min while CAT unit "U" is defined as H_2O_2 consumed/minute.

doi:10.1371/journal.pone.0139831.g002

attenuate the ROS production in hepatocytes. Lipid peroxidation is an indication of oxidative tissue damage caused by ROS. TBARS is a yard stick of lipid-peroxidation. In this study, Pb-acetate treatment significantly increased ($p < 0.01$) TBARS levels as compared to control hepatocytes (Fig 2B). However, AEIA treatment could significantly alleviate ($p < 0.01$) the degree of lipid-peroxidation *in vitro* evidenced by reduction of TBARS level. Pb-acetate intoxication caused a significant ($p < 0.01$) increase in the carbonylation of proteins in hepatocytes (Fig 2B). AEIA treatment could significantly reinstate ($p < 0.05$) the Pb-acetate mediated protein

carbonylation in murine hepatocytes. Cellular antioxidant enzymes and GSH served in cellular defense mechanism during redox stress. In this study, the levels of CAT, SOD, GR, GST, GPx and GSH were reduced ($p < 0.01$) significantly in Pb-acetate treated hepatocytes (Fig 2B). However, AEIA treatment could significantly ($p < 0.05$–0.01) revert the levels of aforementioned redox markers to near normal status. The data set is available in S1 Table.

Effect on intrinsic and extrinsic pathways of cell death. The generation of excessive ROS and subsequent oxidative damage plays a crucial role in the induction of apoptosis. In this study, the incidence of apoptosis during Pb-acetate intoxication and the protective role of AEIA have been evaluated by western blot analysis. Pb-acetate intoxication caused an increase in the expression of mitochondrial Bad protein with concomitant down-regulationof cytosolic Bad protein resulting a significant high ($p < 0.01$) mitochondrial Bad/cytosolic Bad ration over untreated hepatocytes (Fig 3). It indicated translocation of Bad from cytosol to mitochondria. Pb-intoxication significantly reduced the expression of Bcl-2 resulting a significantly high ($p < 0.01$) mitochondrial Bad/Bcl-2 over untreated hepatocytes (Fig 3). Pb-acetate treatment caused significant increase in the expression of cytosolic Cyt C over mitochondrial Cyt C resulting a significantly ($p < 0.01$) high cytosolic Cyt C/mitochondrial Cyt C ratio over unity (Fig 3). The elevated release of Cyt C opened caspase cascade through cleavage of pro-caspases into their respective cleaved and active fractions. In this study, significant increase ($p < 0.01$) in the expressions of cleaved caspases 3 and 9 was observed in the Pb-acetate treated hepatocytes (Fig 3). Immunoblot analysis showed that Pb-acetate exposure increased significantly ($p<0.01$) the expression of Apaf-1 (Fig 3). The aforementioned observation indicated the involvement of intrinsic mediator mediated apoptotic event during Pb-intoxication.

To study the effect of Pb-acetate in extrinsic factors' mediated apoptosis, immunoblot analysis of FAS, Bid and cleaved caspase-8 were performed (Fig 4). Pb-acetate significantly ($p < 0.01$) up-regulated the FAS, Bid and caspase 8 in isolated murine hepatocytes, which indicated the involvement of extrinsic pathway of apoptosis, simultaneously.

However, treatment of the cells with AEIA could significantly ($p < 0.05$–0.01) reciprocate the Pb-mediated alteration in the expressions of transcription proteins involved in the of intrinsic and extrinsic apoptotic pathway.

Effect of AEIA against Pb-acetate-intoxication *in vivo*

Before demonstrating the protective effect of AEIA against Pb-acetate-intoxication *in vivo*, it would be worthy to mention that we have performed a preliminary sub-acute toxicity study of AEIA (100 mg/kg). We have studied the effect of AEIA (100 mg/kg) on haematological, serum biochemical, ROS production, lipid peroxidation, protein carbonyation, the status endogenous antioxidants (anti-oxidant enzymes and GSH). The data has been represented under S1 and S2 Tables. No significant difference was observed in either of studied parameter as compared with normal control. Also we did not find any change in the histological section as compared with normal control (data were not shown).

Haematologial parameters. Results of various treatments on blood Pb content, haematological and serum biochemical parameters have been shown in Table 1. Pb-acetate treatment increased significantly ($p < 0.01$) the blood Pb content. AEIA treatment reduced ($p < 0.05$) significantly the Pb burden in blood. In search of hematological parameters, a substantial reduction ($p < 0.05$) in total erythrocytes count and haemoglobin content was observed in the mice treated with Pb-acetate (group II), while no significant difference in total leucocytes was observed. Treatment of AEIA, however, could significantly ($p < 0.05$) improved haemoglobin content as compared with Pb-acetate treated animals. Pb-acetate-treated mice exhibited substantial increase ($p < 0.01$) in LDH, CK, cholesterol and triglyceride levels. However, treatment

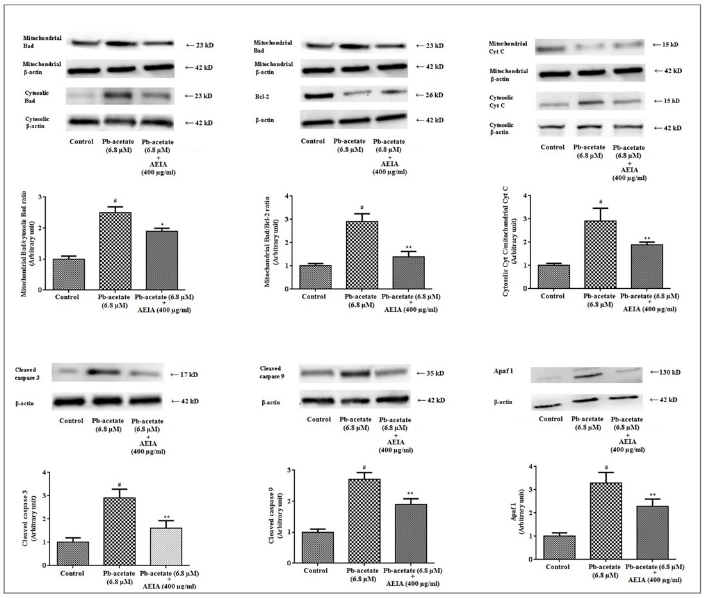

Fig 3. Respective western blot analysis of Bcl-2, Bad, cleaved caspase 9 and 3, Cyt C, and Apaf 1 in the absence (Pb-acetate) and existence of AEIA (Pb-acetate + AEIA) in mouse hepatocytes. The relative band intensities were measured and the normal control band was given an arbitrary value of 1. β-actin was used as a loading protein. Values were expressed as mean ± SE (n = 3). #Values differed (p < 0.01) significantly from normal control. *Values differed (p < 0.05) significantly from Pb-acetate. ** Values differed (p < 0.01) significantly from Pb-acetate control.

doi:10.1371/journal.pone.0139831.g003

of the extract (100 mg/kg) exhibited significant improvements ($p < 0.05$–0.01) in all the aforementioned serum parameters to near normal status.

Results of Pb-accumulation in tissues. The accumulation of Pb in the tissues is thought to be the principle cause of Pb-intoxication. The effect of Pb-acetate treatment in the intracellular Pb-burden has been estimated in kidney, liver, heart, brain and testes of experimental mice (Fig 5). Pb-acetate treatment significantly increased ($p < 0.01$) the Pb-bioaccumulation in the aforementioned organs. The degree of Pb accumulation was found to in the order of kidney > liver > heart > testes > brain. However, AEIA treatment could significantly ($p < 0.01$) prevent intracellular Pb burden in the aforementioned tissues, as compared to Pb-acetate treated mice. Data set is available in S4 Table.

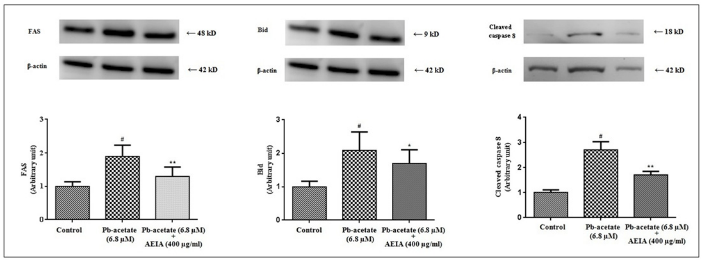

Fig 4. Respective western blot analysis of Fas, Bid and caspase 8 in the absence (Pb-acetate) and existence of AEIA (Pb-acetate + AEIA) in murine hepatocytes. The relative band intensities were measured and the normal control band was given an arbitrary value of 1. β-actin was used as a loading protein. Values are expressed as mean ± SE (n = 3). # Values differed (p < 0.01) significantly from normal control. *Values differed (p < 0.05) significantly from Pb-acetate control. **Values differed (p < 0.05) significantly from Pb-acetate control.

doi:10.1371/journal.pone.0139831.g004

Table 1. Effect on haematological and serum-biochemical parameters in the absence (Pb-acetate) and existence of AEIA (AEIA + Pb-acetate) in mice.

Groups	Haematological and serum biochemical parameters	Values
Normal control	Pb content (μg/ml)	0.05 ± 0.006
Toxic control (Pb-acetate)		0.95 ± 0.07#
Pb-acetate + AEIA		0.72 ± 0.09*
Normal control	Total erythrocyte count (x10^{6}/mm^{3})	5.46 ± 0.67
Toxic control (Pb-acetate)		3.45 ± 0.33$
Pb-acetate + AEIA		4.77 ± 0.54
Normal control	Haemoglobin (g/dl)	9.11 ± 0.95
Toxic control (Pb-acetate)		6.15 ± 0.72$
Pb-acetate + AEIA		8.76 ± 0.50*
Normal control	Total leucocyte count (x10^{3}/mm^{3})	6.05 ± 0.46
Toxic control (Pb-acetate)		5.15 ± 0.55
Pb-acetate + AEIA		5.77 ± 0.82
Normal control	Lactate dehydrogenase (U/l)	31.18 ± 1.84
Toxic control (Pb-acetate)		52.31 ± 2.25#
Pb-acetate + AEIA		41.16 ± 1.98**
Normal control	Creatinine kinase (IU/ mg protein)	192.67 ± 9.10
Toxic control (Pb-acetate)		272.50 ± 12.05#
Pb-acetate + AEIA		232.77 ± 11.23*
Normal control	Cholesterol (mg/dl)	188.12 ± 9.65
Toxic control (Pb-acetate)		276.33 ± 11.04#
Pb-acetate + AEIA		229.45 ± 13.11**
Normal control	Triglycerides (mg/dl)	121.50 ± 7.24
Toxic control (Pb-acetate)		208.78 ± 9.34#
Pb-acetate + AEIA		178.95 ± 8.02*

Values are expressed as mean ± SE, (6 mice per group).

$ Values significantly differed from normal control (p < 0.05).

Values differ significantly from normal control (p < 0.01).

*Values significantly differed from Pb-acetate control (p < 0.05).

** Values significantly differed from Pb-acetate control (p < 0.01).

doi:10.1371/journal.pone.0139831.t001

Effects on ATP level and DNA fragmentation. The effect of Pb-intoxication in DNA fragmentation and ATP levels were shown in Fig 5 (Data set is available in S4 Table). In this study, Pb-acetate treatment significantly increased ($p < 0.01$) cellular DNA disintegration in liver, kidney, brain, heart, and testes as compared to normal control. However, treatment with AEIA could significantly reverted ($p < 0.05$–0.01) the cellular DNA fragmentation in the aforementioned tissues. Cellular ATP level is an indication of status of cellular events. The increased ($p < 0.01$) levels of ATP in the selected tissues indicated the induction of apoptosis during Pb-intoxication. However, extract treatment could significantly ($p < 0.05$–0.01) restore cellular ATP levels near to normalcy.

Effect on ROS, Protein-carbonylation, lipid peroxidation, co-enzyme Q and redox status of the tissues. Pb-intoxication significantly enhanced ($p < 0.01$) intercellular ROS-generation, lipid peroxydation and protein-carbonylation in the hepatic, renal, cardiac, cerebral and testicular tissues (Fig 6). Data set is available in S5 Table. AEIA treatment, however, could significantly reduce the extent of ROS production ($p < 0.01$), lipid peroxydation ($p < 0.05$–0.01) and protein carbonylation ($p < 0.05$–0.01) in the aforementioned tissues. The results of various

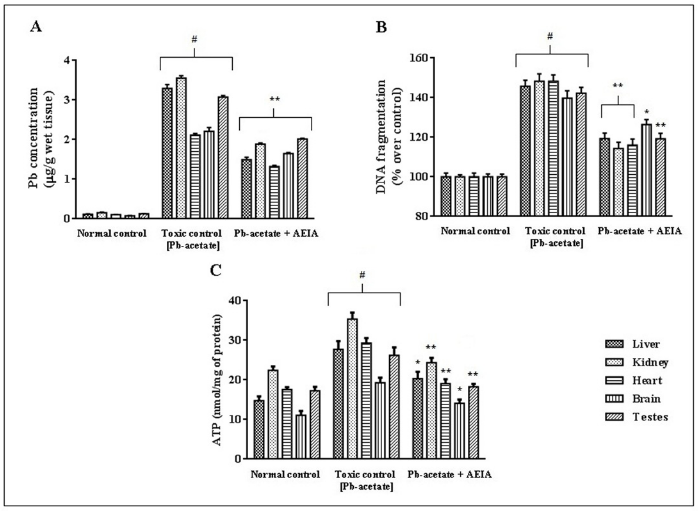

Fig 5. Effect on Pb-bioaccumulation (A), DNA fragmentation (B) and ATP level (C) in the absence (Pb-acetate) and existence of AEIA (Pb-acetate +AEIA) in liver, kidney, heart, brain and testes in experimental mice. Values are denoted as mean ± SE (n = 6). # Values significantly differed ($p < 0.01$) from normal control. *Values significantly differed ($p < 0.05$) from differed Pb-acetate control. ** Values significantly differed ($p < 0.01$) from differed Pb-acetate control.

doi:10.1371/journal.pone.0139831.g005

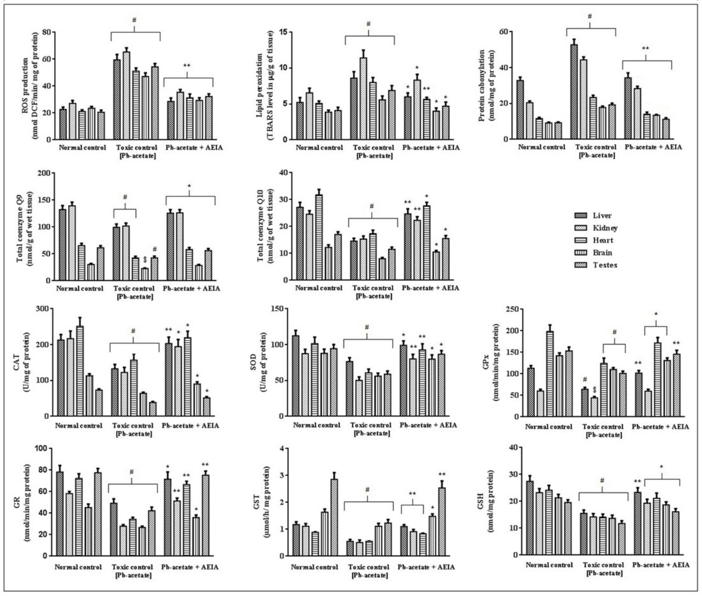

Fig 6. Effect on ROS production, lipid peroxidation, protein carbonylation, co-enzyme Q9, co-enzyme Q10, CAT, SOD, GPx, GR, GST and GSH in the absence (Pb-acetate) and existence of AEIA (Pb-acetate +AEIA) in experimental mice. Values are denoted as mean ± SE (n = 6). $Values significantly differed ($p < 0.05$) from differed Pb-acetate control. # Values significantly differed ($p < 0.01$) from normal control. * Values significantly differed ($p < 0.05$) from differed Pb-acetate control. ** Values significantly differed ($p < 0.01$) from differed Pb-acetate control. SOD unit, "U" is defined as µ-moles inhibition of NBT-reduction/min while CAT unit "U" is defined as H_2O_2 consumed/minute.

doi:10.1371/journal.pone.0139831.g006

treatments on mitochondrial-ubiquinols (coenzymes Q_9 & Q_{10}) in liver, kidney, brain, heart, and testes have been estimated (Fig 6). Total co-enzyme Q_9 and Q_{10} levels in the tissues were significantly ($p < 0.01–0.05$) decreased in Pb-acetated treated groups (group II). Treatment with AEIA significantly elevated Q_9 levels in liver and kidney, heart, brain and testes ($p < 0.05$) as compared with toxic control group. On other hand, co-enzyme Q_{10} levels have been found to be significantly elevated in hepatic ($p < 0.01$), renal ($p < 0.01$), cardiac ($p < 0.05$), brain ($p < 0.05$) and testicular ($p < 0.05$) tissues following AEIA treatment. In search of protective role of AEIA against Pb-toxicity, the effects on tissue redox markers viz. antioxidant enzymes and reduced glutathione were estimated (Fig 6). Data set is available in S6 Table. Pb-acetate treatment significantly ($p < 0.05–0.01$) decreased the levels of tissue antioxidant enzymes namely CAT, SOD, GR, GST and GPx in the selected tissues of experimental mice. In search of effect on tissue GSH levels, Pb-acetate treatment decreased GSH levels significantly ($p < 0.01$) in the selected tissues of experimental mice. However, AEIA treatment could significantly ($p < 0.05–0.01$) revert the Pb-acetate mediated reduction of tissue redox markers near to normal status.

Histological assessment. The histological sections of livers of mice have been depicted in Fig 7A (x 100) and B (x 400). The liver section of Pb-acetate-intoxicated mice exhibited diffuse portal veins, inflamed hepatocytes with infiltrating leukocytes and lipid deposition when compared with the liver sections of normal mice. Histo-quantification revealed a significant elevation of % of lipid deposition ($p < 0.01$), % of the area of inflamed hepatocytes ($p < 0.01$) and the % of area of portal veins ($p < 0.05$) in Pb-intoxicated liver sections (Fig 7C–7E). AEIA treatment could significantly ($p < 0.01$) revert the lipid deposition and hepatic inflammation to near normal status, however, extract treatment could insignificantly ($p < 0.05$) arrest the dilation of portal vein. The histological sections of kidneys of mice have been shown in Fig 8A (x 100) and B (x 400). The kidney sections of Pb-acetate control mice exhibited thickening of bowman's capsules and cellular damage with cloudy appearance of tubules when compared with normal control animals. Histo-quantification revealed that Pb-intoxication caused significant thickening in capsular space ($p < 0.01$) and cloudy swelling of renal tubules ($p < 0.01$) (Fig 8C and 8D). Treatment with AEIA significantly ($p < 0.05–0.01$) reduced the Pb-induced pathological changes and restores the histology near to normalcy. Histological sections of hearts have been revealed in Fig 9A (x 100) and 9B (x 400). Pb-intoxication caused significant damage of the interstitial tissues ($p < 0.01$) and muscle replacement by adipose tissues ($p < 0.01$) (Fig 9C and 9D). AEIA treatment could significantly ($p < 0.01$) arrest the damage in cardiac section. The histological sections of brains have been represented in Fig 10A (x 100) and 10B (x 400). Pb-intoxicated mice exhibited a significant increase in % of the vacuolated area of degenerated tissues ($p < 0.01$) and diffused edema ($p < 0.01$) as compared to normal control mice (Fig 10C and 10D). However, treatment with AEIA could significantly ($p < 0.01$) reinstate Pb-mediated toxic manifestation in brain. The sections of testes have been shown in Fig 11A (x 100) and 11B (x 400). The testes segment of Pb-acetate treated mice exhibited substantial disintegration of seminiferous tubules with loss of spermatogenic cells. The histoquantification of testicular sections were scored with Jhonsen score (Fig 11C). A significant ($p < 0.01$) reduction of Jhonsen score was observed in Pb-intoxicated mice. However, AEIA treatment could significantly ($p < 0.01$) reinstate the Jhonsen score and restore the histology near to normalcy.

Discussion

Pb is a toxic heavy metal of great occupational importance. Pb-poisoning principally arises from Pb contaminated air, dust and soils by the Pb based paints, fertilizers, automobiles,

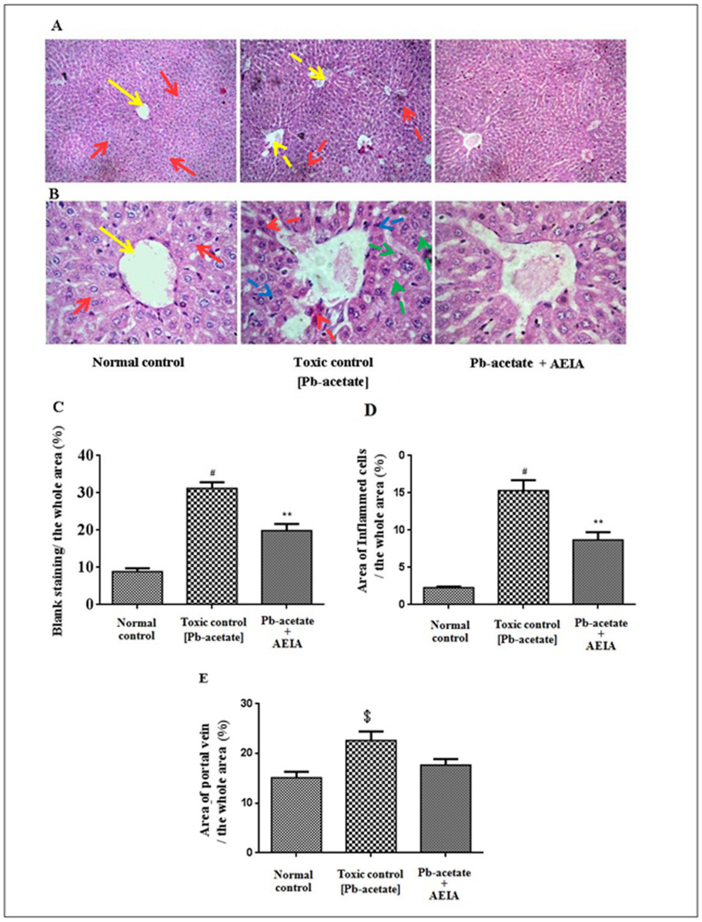

Fig 7. Histological sections 100 x (A) and 400 x (B) of livers of experimental mice in the absence (Pb-acetate) and existence of AEIA (Pb-acetate + AEIA). Untreated mice were kept as normal control to compare the structural changes caused by Pb-acetate. Yellow and red arrows represent normal portal vein and hepatocytes, respectively; dotted arrows represent the Pb-acetate mediated structural changes of portal vein (yellow), hepatocytes (red) with infiltrating leukocytes (blue) and lipid deposition (green). C. Hepatic lipid droplets accumulation was presented as the percentage of the blank area relative to the whole area of the photomicrograph (100 x, randomly selected area devoid of portal vein were selected). D. The incidence of inflammation was presented as the percentage of the inflamed hepatocytes region relative to the whole area of the photomicrograph (100 x, randomly selected area in devoid of portal vein were selected). E. The structural change of portal vein was represented as percentage of the blank area relative to the whole area of the photomicrograph (400 x, randomly selected areas containing one portal vein were selected). Values are expressed as mean ± SE, (n = 60). $^{\$}$ Values significantly differed from normal control ($P < 0.05$). $^{\#}$ Values differ significantly from normal control ($p < 0.01$). **Values significantly differed from Pb-acetate control ($P < 0.01$).

doi:10.1371/journal.pone.0139831.g007

cosmetics, batteries etc. [22]. Despite the use of Pb has been restricted in many fields of its applications, however, the consumption of Pb is alarmingly exceeding over past decades [1]. Pb is a multi-target toxicant exerts toxic manifestation by oxidative free radicals mediated disruption of delicate pro- and anti- oxidant balance existing in mammalian cells. Generation of ROS is known to cause damage to cellular biomolecules mainly lipids, proteins and nucleic acids [21]. In this study, a substantial increase in generation of ROS was observed in hepatocytes as well different organs after Pb exposure. An increase in the Pb bioaccumulation is directly associated with the ROS production.

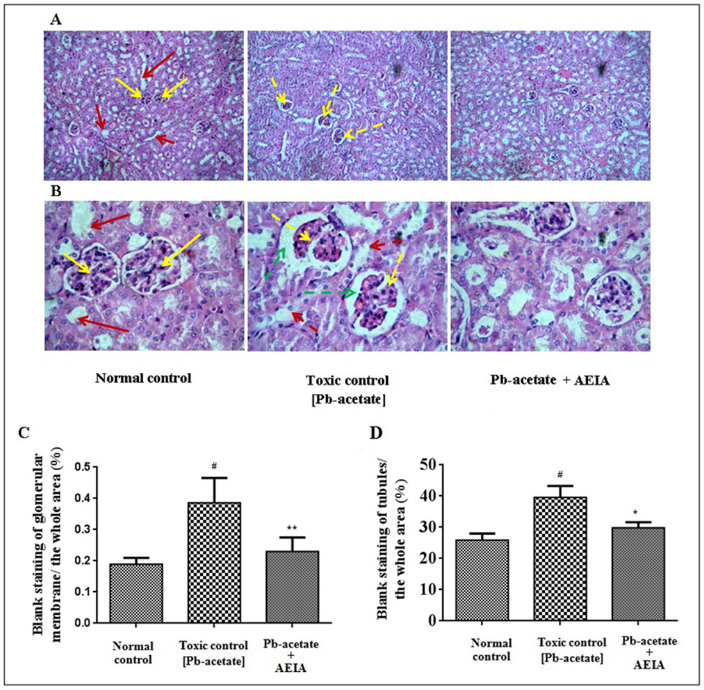

Fig 8. Histological sections 100 x (A) and 400 x (B) of kidneys of experimental mice in absence (Pb-acetate) and presence of AEIA (Pb-acetate + AEIA). Untreated mice were kept as normal control to compare the structural changes caused by Pb-acetate. Yellow and red arrows represent normal glomerulus structure and renal tubules, respectively; dotted arrows represent the Pb-acetate mediated glomerular hypercellularity (yellow) and capsular space thickening (green), and cloudy appearance of renal tubules (red). C. The widening of capsular space was represented as percentage of the blank area relative to the whole area of the photomicrograph (400 x, randomly selected areas containing one glomerulus were chosen). D. The dilation of renal tubules was represented as percentage of the blank area relative to the whole area of the photomicrograph (100 x, randomly selected areas devoid of any glomerulus were selected). Values are expressed as mean ± SE, (n = 60). # Values differ significantly from normal control ($p < 0.01$).* Values significantly differed from Pb-acetate control ($P < 0.05$).

doi:10.1371/journal.pone.0139831.g008

ROS production induced apoptosis mediated by both intrinsic and extrinsic mechanisms. Apoptotic pathway is executed by some pro-apoptotic (Bad) and anti-apoptotic (Bcl-2) proteins and caspases. The anti-apoptotic members of Bcl-2 family could restrict cytosolic cytochrome C release [13]. The proapoptotic members of Bcl-2 family counteract with the cytoprotective effect of anti-apoptotic Bcl-2 family proteins and thereby promoting cytochrome C release in cytosol [33, 34]. The initiation of apoptosis is mediated by translocation of proapoptotic Bad to mitochondria followed by down-regulation of antiapoptotic, Bcl-2, which leads to an up-regulation of cytosolic Cyt C over mitochondrial Cyt C. Enhanced discharge of cytochrome C promotes cleavage of caspases and thereby induces apoptosis by intrinsic pathways. On other side, Fas-mediated activation of Bid and cleavage of pro-caspase 8 into its cleaved fraction induce apoptosis by extrinsic pathway [9]. In the present study, immunoblot analyses confirmed the participation of both extrinsic and intrinsic path-ways of apoptosis following Pb-acetate exposure *in vitro*, which causes a significant reduction in the viability of murine hepatocytes. The flow cytometric analysis confirmed that the incidence of apoptosis during Pb-acetate intoxication, however, AEIA could significantly counteract with Pb-acetate mediated apoptosis. Many of earlier reports corroborated the direct involvement of ROS generation in the process of apoptosis via alteration of transcription of apoptotic proteins within the cells [9, 21, 24, 32, 35]. The excessive production of ROS coupled with the depletion of

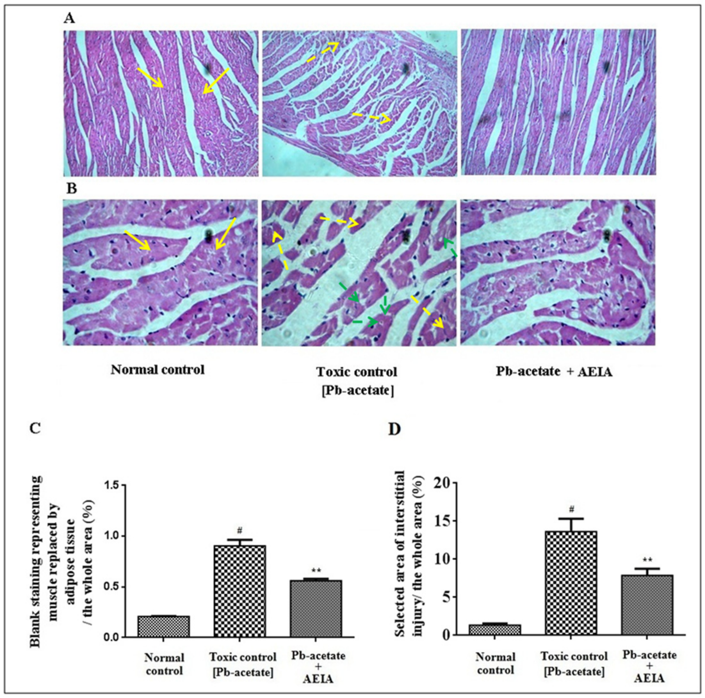

Fig 9. Histological sections 100 x (A) and 400 x (B) of hearts of experimental mice in the absence (Pb-acetate) and existence of AEIA (Pb-acetate + AEIA). Untreated mice were kept as normal control to compare the structural changes caused by Pb-acetate. Yellow arrows denote normal radiating pattern of cardiac muscle; yellow dotted arrows indicate excessive degradation in cardiac muscles and green dotted arrows represent replacement of muscle by adipose tissues during Pb-intoxication. C. The blank selected area represented the extent of cardiac muscle replaced by adipose tissue relative to the whole area of the photomicrograph (400 x, randomly selected areas). D. The selected area of interstitial injury relative to the whole area of the photomicrograph (400 x, randomly selected areas). Values are expressed as mean ± SE, (n = 60). $^{\#}$ Values differ significantly from normal control ($p < 0.01$). ** Values significantly differed from Pb-acetate control ($p < 0.01$).

doi:10.1371/journal.pone.0139831.g009

endogenous antioxidant defense systems, therefore, necessitates the supplement/therapy with exogenous antioxidants to counter act with excess of ROS [32, 35]. In this study, we found that AEIA could capable to counteract with Pb-acetate mediated generation of cellular ROS. Considering the integral relationship between ROS and apoptotic incidence, it would be presumed that the AEIA may counteract with Pb-induced apoptosis through scavenging of ROS.

The Pb-induced oxidative stress mechanism includes the effect of Pb on membrane lipids, cellular functional proteins and DNA. The presence of = bonds in fatty acids of cell membranes weakens the adjacent C-/H bonds. Later ensures easier removal of H during oxidative stress and promotes the progress of lipid peroxidation reaction. Therefore, the polyunsaturated fatty acids containing more than 2 double bonds are more susceptible to lipid peroxidation. Pb-mediated oxidative stress could also affect the carbonylation of cellular proteins and the signal transduction process. Earlier reports revealed that Pb could cause oxidative damage of DNA. The mechanism is less conclusive, however, the alkalating effect of 4,5 di-oxo-valeric acid, the end oxidation product of δ-amino-levulinic acid which accumulates during Pb-intoxication has been proposed to be the mechanism [36]. The protective effect of AEIA may be due to its antioxidant effect via scavenging ROS.

Pb has strong affinity with sulfhydryl and amide groups, thereby inhibiting many enzymes namely CAT, GR, SOD and GPx etc. Previous report revealed that CAT, GR, SOD and GPx

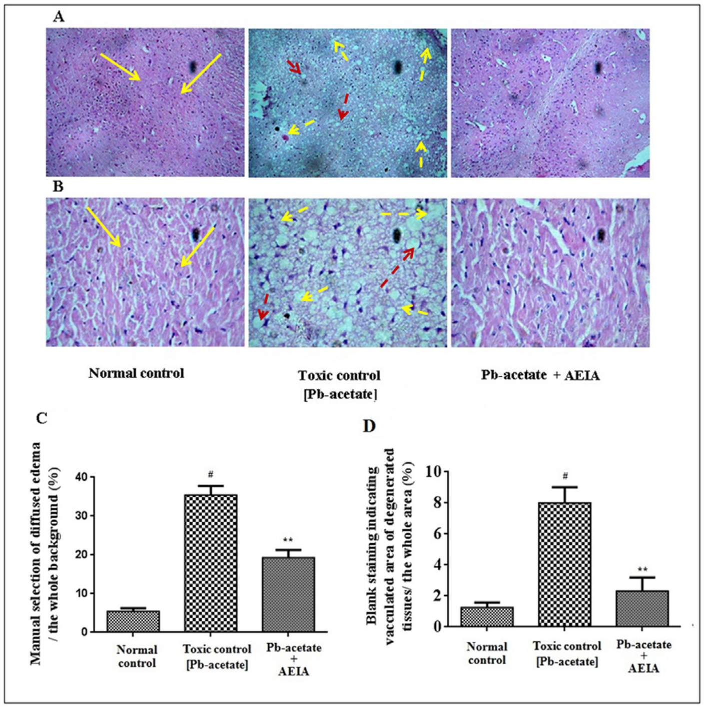

Fig 10. Histological sections 100 x (A) and 400 x (B) of brains of experimental mice in the absence (Pb-acetate) and existence of AEIA (Pb-acetate + AEIA). Untreated mice were kept as normal control to compare the structural changes caused by Pb-acetate. Yellow arrows represent normal normal cyto-architecture of brain; dotted arrows represent the Pb-acetate mediated development of vacuolated area of degenerated tissues (yellow) and diffused edema (red). C. The manually selected area of diffused edema relative to the whole area of the photomicrograph (400 x, randomly selected areas). D. The blank staining representing vacuolated area of degenerated tissues relative to the whole area of the photomicrograph (400 x, randomly selected areas). Values are expressed as mean ± SE, (n = 60). # Values differ significantly from normal control ($p < 0.01$). ** Values significantly differed from Pb-acetate control ($p < 0.01$).

doi:10.1371/journal.pone.0139831.g010

are potential targets for Pb [37]. Pb mediated imbalance in various trace factors would be responsible for the case [37]. The extracts may exert their effect by diverse mechanisms, mainly by quenching, addition and/or recombination of free radical and/or promoting clearance of Pb from the tissues. In this study, we observed that AEIA could significantly inhibited ROS production also promote clearance of Pb from the organs.

Abnormalities in haematological and serum biochemical parameters are the earlier diagnostic features of any pathological incidence within the body. In this study, increased serum levels of membrane bound enzymes indicated the cellular damage during Pb-intoxication. The higher levels of serum lipids indicate increased lipogenesis and/or decreased clearance of lipoproteins during Pb toxicity, which is in accordance to previous observation [22]. Pb-intoxication also affects the reduction of erythrocytes and total haemoglobin contents, which could be associated with excess of binding of Pb with circulating erythrocytes [38]. The treatment of AEIA could significantly rescue the haematological and serum biochemical parameters which supported the prophylactic role of the extract.

Finally, histological assessments showed that Pb-acetated caused abnormal histological changes in the sections of liver, kidney, heart, brain and testes, which has been significantly attenuated following AEIA treatment to near-normal status.

Existing literature revealed that, bioaccumulation of Pb causes generation of excessive ROS which could cause redox imbalance and participate pivotal role in Pb-toxicity. Therefore, the

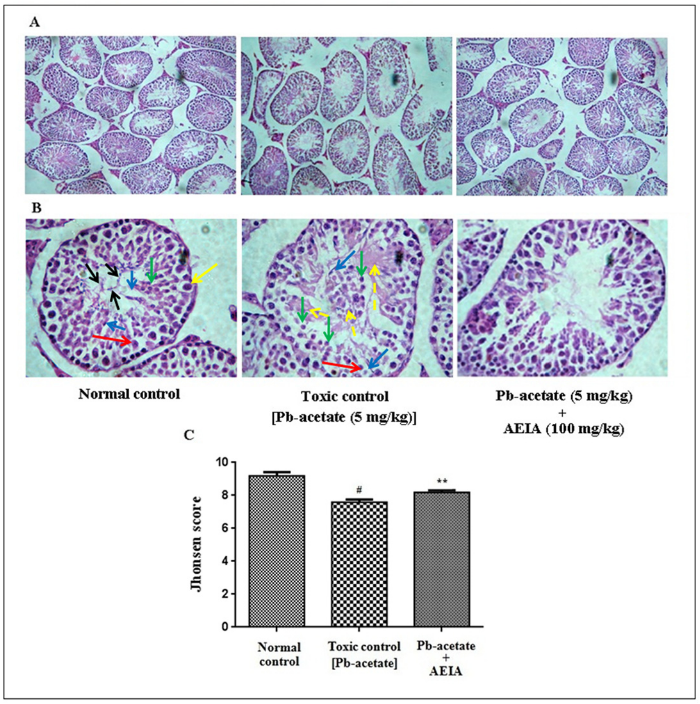

Fig 11. Histological sections 100 x (A) and 400 x (B) of testes of experimental mice in the absence (Pb-acetate) and existence of AEIA (Pb-acetate + AEIA). Untreated mice were kept as normal control to compare the structural changes caused by Pb-acetate. The section of normal control illustrating the typical structure of seminiferous tubule at Johnsen score of ~ 9–10 showing all stages of spermatogenesis. Yellow arrow represents spermatogonia near to the basement membrane; Red arrow represents primary spermatocytes; Green arrow represents round spermatids; Blue arrows represent elongated spermatids; Black arrows represent mature spermatozoa. Pb-acetate intoxicated group exhibited seminiferous tubule showing Johnsen score of ~ 6–7. Tubule contains primary spermatocytes (red arrow); round (green arrows) and elongated (blue arrows) spermatids, and cloudy abnormal spermatozoa (yellow dotted). AEIA treated section exhibited Johnsen score of ~ 8–9. C. The Johnsen score was determined (400 X, containing one seminiferous tubule). Values are expressed as mean ± SE, (n = 60). # Values differ significantly from normal control (p < 0.01). ** Values significantly differed from Pb-acetate control (P < 0.01).

doi:10.1371/journal.pone.0139831.g011

agents promoting Pb clearance and/or scavenging ROS would participate in counteracting Pb-poisoning. In present investigation, excessive generation of ROS following Pb exposure caused oxidative tissue damage via apoptosis and hampered cellular redox defence system (Fig 12). Experimental data revealed that the treatment of the edible extract of the edible aerial parts of *I. aquatica* could cause significant reversal of Pb-acetate induced toxic manifestations in both *in vivo* and *in vitro* systems. The extract would offer the protective effect via counteracting with Pb mediated oxidative stress and/or promoting the elimination of Pb by chelating (Fig 12). Phytochemical studies revealed presence of flavonoids, phenolic compounds and ascorbic acid in the test extracts. Substantial quantities of aforementioned dietary antioxidants [9,22,24] could contribute in the ROS scavenging during Pb-intoxication. Besides, saponins and flavonoids are known possess Pb chelating property [39,40]. Therefore, dietary antioxidants would serve as primary healthcare against ROS mediated Pb poisoning and the diet comprising *I. aquatica* would serve as useful clinical medicine against Pd toxicity. Present study is continued to isolate scaffolds for the overall protective effect of *I. aquatica*.

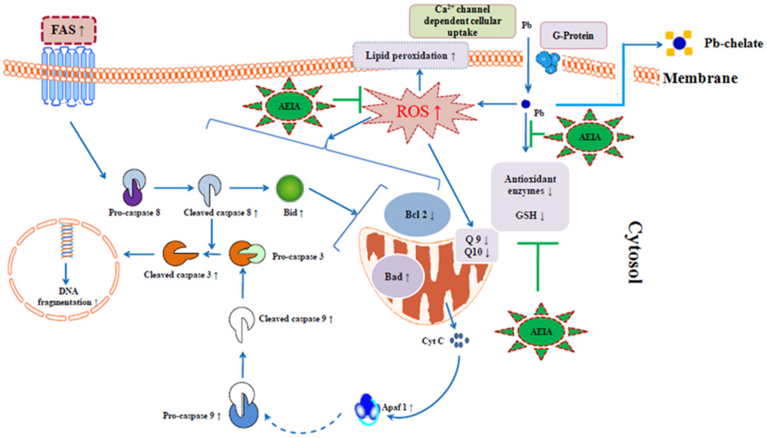

Fig 12. Schematic presentation of probable protective mechanism of AEIA against Pb toxicity. The blue arrows indicate the cellular events involved in Pb pathogenesis. The green colour line denotes the site of action of AEIA.

doi:10.1371/journal.pone.0139831.g012

Supporting Information

S1 Table. Effect on antioxidant parameters in absence (Pb-acetate) and presence of AEIA (AEIA + Pb-acetate) in isolated mice hepatocytes.
(DOCX)

S2 Table. Effect of AEIA (100 mg/kg, p.o.) on haematological parameters of experimental mice.
(DOCX)

S3 Table. Effect of AEIA (100 mg/kg, p.o.) on ROS production, lipid peroxidation, protein carbonylation, antioxidant enzymes and GSH levels in liver, kidney, heart, brain and testes of experimental mice.
(DOCX)

S4 Table. Effect on antioxidant enzymes and GSH levels in liver, kidney, heart, brain and testes in absence (Pb-acetate) and presence of AEIA (AEIA + Pb-acetate) in mice.
(DOCX)

S5 Table. Effect on ROS production, lipid peroxidation, protein carbonylation and Co-enzymes Qs levels in liver, kidney, heart and brain in absence (Pb-acetate) and presence of AEIA (AEIA + (Pb-acetate) in mice.
(DOCX)

S6 Table. Effect on antioxidant enzymes and GSH levels in liver, kidney, heart, brain and testes in absence (Pb-acetate) and presence of AEIA (AEIA + Pb-acetate) in mice.
(DOCX)

Acknowledgments

The financial support of the Council for Scientific and Industrial Research, New Delhi, India is gratefully acknowledged through Senior Research Fellowship to Mr. Tarun K. Dua [Council for Scientific and Industrial Research-senior research fellowship Ref. No.: 09/096(773)/2013-EMR-I dated 15th March, 2013]. Authors are grateful to Jadavpur University, Kolkata, India for providing necessary facilities for this study. All authors are thankful to Dr. Avinava

Mukherjee for his kind help for histo-quantification analysis. Finally, the authors would like to say thanks to the reviewers for their time and valuable comments to improve the quality of this manuscript.

Author Contributions

Conceived and designed the experiments: SD HZEJ MZUH. Performed the experiments: SD TKD RK SD SB SJ NB. Analyzed the data: SD. Contributed reagents/materials/analysis tools: SD TKD. Wrote the paper: SD HZEJ MZUH.

References

1. Hsu P-C, Guo YL. Antioxidant nutrients and lead toxicity. Toxicol. 2002; 180:33–44.
2. De Silva PE. Determination of lead in plasma and studies on its relationship to lead in erythrocytes. Br J Ind Med. 1981; 38:209–17. PMID: 6791682
3. Sharama RP, Street JC. Public health aspects of toxic heavy metals in animal feeds. J Am Vet Med Assoc. 1980; 177:149–53. PMID: 7429947
4. Humphreys DJ. Effects of exposure to excessive quantities of lead on animals. Br Vet J. 1991; 147:18–30. PMID: 2018915
5. Bressler J, Kim KA, Chakraborti T, Goldstein G. Mechanism of lead neurotoxicity. Neurochem Res. 1999; 24:595–600. PMID: 10227691
6. Khalil-Manesh F, Gonick HC, Weiler EJ, Prins B, Weber MA, Purdy R. Lead-induced hypertension: possible role of endothelial factors. Am J Hypertens. 1993; 6:723–9. PMID: 8110424
7. Lancranjan I, Popescu JI, Gavenescu O, Klepsch I, Serbanescu M. Reproductive ability of workmen occupationally exposed to lead. Arch Environ Health 1975; 30:396–401. PMID: 1155972
8. Frei B, Higdon J. Antioxidant activity of tea polyphenols *in vivo*: evidence from animal studies. J Nutr. 2003; 133:3275–84.
9. Dua TK, Dewanjee S, Gangopadhyay M, Khanra R, Zia-Ul-Haq M, De Feo V. Ameliorative effect of water spinach, *Ipomoea aquatic* (Convolvulaceae), against experimentally induced arsenic toxicity. J Transl Med. 2015; 13:81. doi: 10.1186/s12967-015-0430-3 PMID: 25890105
10. Chopra RN, Nayar SL, Chopra IC. Glossary of Indian medicinal plants. New Delhi, India: Council of Scientific & Industrial Research. 1956.
11. Public Health Service (PHS). Public health service policy on humane care and use of laboratory animals. Washington, DC. US Department of Health and Human Services; Available from Office for Protection from Research Risks, Building 31, Room 4B09, NIII, Bethesda, MD 20892. 1986.
12. Sarkar K, Sil PC. A 43 kDa protein from the herb *Cajanus indicus* L. protects thioacetamide induced cytotoxicity in hepatocytes. Toxicol In Vitro 2006; 20:634–40. PMID: 16403433
13. Pal S, Pal PB, Das J, Sil PC. Involvement of both intrinsic and extrinsic pathways in hepatoprotection of arjunolic acid against cadmium induced acute damage *in vitro*. Toxicol. 2011; 283:129–39.
14. Mansoor TA, Borralho PM, Dewanjee S, Mulhovo S, Rodrigues CPM, Ferreira MJU. Monoterpene bisindole alkaloids, from the African medicinal plant *Tabernaemontana elegans*, induce apoptosis in HCT116 human colon carcinoma cells. J Ethnopharmacol. 2013; 149:463–70. doi: 10.1016/j.jep.2013.06.051 PMID: 23872252
15. Kim J, McCarte RJM, Yu BP. Influence of age, exercise and dietary restriction on oxidative stress in rats. Aging Clin Exp Res. 1996; 8:123–9.
16. LeBel CP, Bondy SC. Sensitive and rapid quantitation of oxygen reactive species formation in rat synaptosomes. Neurochem Int. 1990; 17:435–40. PMID: 20504643
17. Ohkawa H, Ohishi N, Yagi K. Assay for lipid peroxides in animal tissues by thiobarbituric acid reaction. Anal. Biochem. 1979; 95:351–8. PMID: 36810
18. Uchida K, Stadtman ER. Covalent attachment of 4-hydroxynonenal to glyceraldehydes-3-phosphate dehydrogenase. A possible involvement of intra- and intermolecular cross-linking reaction. J Bio Chem. 1993; 268:6388–93.
19. Ghosh J, Das J, Manna P, Sil PC. Protective effect of the fruits of *Terminalia arjuna* against cadmium-induced oxidant stress and hepatic cell injury via MAPK activation and mitochondria dependent pathway. Food Chem. 2010; 123:1062–75.
20. Hissin PJ, Hilf R. A fluorometric method for the determination of oxidized and reduced glutathione in tissues. Anal Biochem. 1973; 74:214–6.

21. Khanra R, Dewanjee S, Dua TK, Sahu R, Gangopadhyay M, De Feo V, et al. *Abroma augusta* L. (Malvaceae) leaf extract attenuates diabetes induced nephropathy and cardiomyopathy via inhibition of oxidative stress and inflammatory response. J Transl Med. 2015; 13:6. doi: 10.1186/s12967-014-0364-1 PMID: 25591455
22. Dewanjee S, Sahu R, Karmakar S, Gangopadhyay M. Toxic effects of lead exposure in wistar rats: involvement of oxidative stress and the beneficial role of edible jute (*Corchorus olitorius*) leaves. Food Chem Toxicol. 2013; 55:78–91. doi: 10.1016/j.fct.2012.12.040 PMID: 23291325
23. Zhang Y, Aberg F, Appelkvist EL, Dallner G, Ernster L. Uptake of dietary coenzyme Q supplement is limited in rats. J Nutrition 1995; 125:446–53.
24. Dewanjee S, Gangopadhyay M., Sahu R, Karmakar S. Cadmium induced pathophysiology: Prophylactic role of edible jute (Corchorus olitorius) leaves with special emphasis on oxidative stress and mitochondrial involvement. Food Chem Toxicol. 2013; 60:188–98. doi: 10.1016/j.fct.2013.07.043 PMID: 23891759
25. Jensen EC. Quantitative analysis of histological staining and Fluorescence Using ImageJ. Anat Rec. 2013; 296:378–81.
26. Schneider CA, Rasband WA, Eliceiri KW. NIH Image to ImageJ: 25 years of image analysis. Nat Methods 2012; 9:671–5. PMID: 22930834
27. Dongdong WU, Zheng N, Qi K, Cheng H, Sun Z, Gao B, et al. Exogenous hydrogen sulfide mitigates the fatty liver in obese mice through improving lipid metabolism and antioxidant potential. Med Gas Res. 2015; 5:1. doi: 10.1186/s13618-014-0022-y PMID: 25606341
28. Rangan GK, Tesch GH. Quantification of renal pathology by image analysis. Nephrol. 2007; 12:553–8.
29. Ruegg MA, Meinen S. Histopathology in hematoxylin & Eosin stained muscle sections. Sop no. MDC1A_M.1.2.004, Version 1.0. 2014.
30. Defigueiredo RJP, Cummings TBJ, Mundkur P, Cotman CW. Color image analysis in neuroanatomical research: application to senile plaque subtype quantification in Alzheimer's disease. Neurobiol Aging 1995; 16:211–23. PMID: 7777139
31. Saeed K, Tahir M, Lone KP. Effect of Phoenix dactyliferia (date palm) pit powder on nicotine induced spermatotoxicty in adult albino mice. J Pak Med Assoc. 2015; 65: 43–8. PMID: 25831673
32. Bouayed J, Bohn T. Exogenous antioxidants-double-edged swords in cellular redox state health beneficial effects at physiologic doses versus deleterious effects at high doses. Oxid Med Cell Longev. 2010; 3:228–37. PMID: 20972369
33. Dua TK, Dewanjee S, Khanra R, Bhattacharya N, Bhaskar B, Zia-Ul-Haq M, et al. The effects of two common edible herbs, Ipomoea aquatica and Enhydra fluctuans, on cadmium-induced pathophysiology: a focus on oxidative defence and anti-apoptotic mechanism. J Transl Med. 2015; 13:145.
34. Hengartner MO. The biochemistry of apoptosis. Nature 2000; 407:770–6. PMID: 11048727
35. Kannan K, Jain SK. Oxidative stress and apoptosis. Pathophysiol. 2000; 7:153–63.
36. Douki T, Onuki J, Medeiros MH, Bechara EJ, Cadet J, Di Mascio P. DNA alkylation by 4,5-dioxovaleric acid, the final oxidation product of 5-aminolevulinic acid. Chem Res Toxicol. 1998; 11:150–7. PMID: 9511907
37. Gelman BB, Michaelson IA, Bus JS. The effect of lead on oxidative hemolysis and erythrocyte defense mechanisms in the rat. Toxicol Appl Pharmacol. 1978; 45:119–29. PMID: 694930
38. Jackie T, Haleagrahara N, Chakravarthi S. Antioxidant effects of *Etlingera elatior* flower extract against lead acetate—induced perturbations in free radical scavenging enzymes and lipid peroxidation in rats. BMC Res Notes 2011; 4:67. doi: 10.1186/1756-0500-4-67 PMID: 21414212
39. Flora G, Gupta D, Tiwari A. Toxicity of lead: A review with recent updates. Interdiscip Toxicol. 2012; 5:47–58. doi: 10.2478/v10102-012-0009-2 PMID: 23118587
40. Hong KJ, Tokunaga S, Ishigami Y, Kajiuchi T. Extraction of heavy metals from MSW incinerator fly ash using saponins. Chemosphere 2000; 41:345–52. PMID: 11057596
